# Identification of Selection Signals on the X-Chromosome in East Adriatic Sheep: A New Complementary Approach

**DOI:** 10.3389/fgene.2022.887582

**Published:** 2022-04-11

**Authors:** Mario Shihabi, Boris Lukic, Vlatka Cubric-Curik, Vladimir Brajkovic, Milan Oršanić, Damir Ugarković, Luboš Vostry, Ino Curik

**Affiliations:** ^1^ Department of Animal Science, Faculty of Agriculture, University of Zagreb, Zagreb, Croatia; ^2^ Department for Animal Production and Biotechnology, Faculty of Agrobiotechnical Sciences Osijek, J.J. Strossmayer University of Osijek, Osijek, Croatia; ^3^ Department of Forest Ecology and Silviculture, Faculty of Forestry and Wood Technology, University of Zagreb, Zagreb, Croatia; ^4^ Department of Genetics and Breeding, Faculty Agrobiology, Food and Natural Resources, Czech University of Life Sciences, Prague, Czechia

**Keywords:** haplotype richness drop, integrated haplotype score, number of segregating sites by length, runs of homozygosity, selection signals, sheep, X-chromosome

## Abstract

Sheep are one of the most important livestock species in Croatia, found mainly in the Mediterranean coastal and mountainous regions along the East Adriatic coast, well adapted to the environment and mostly kept extensively. Our main objective was therefore to map the positive selection of the X-chromosome (18,983 SNPs that passed quality control), since nothing is known about the adaptation genes on this chromosome for any of the breeds from the Balkan cluster. Analyses were performed on a sample of eight native Croatian breeds (101 females and 100 males) representing the East Adriatic metapopulation and on 10 mouflons (five females and males), all sampled in Croatia. Three classical within-population approaches (extreme Runs of Homozygosity islands, integrated Haplotype Score, and number of Segregating Sites by Length) were applied along with our new approach called Haplotype Richness Drop (HRiD), which uses only the information contained in male haplotypes. We have also shown that phylogenetic analyses, such as the Median-joining network, can provide additional information when performed with the selection signals identified by HRiD. Our new approach identifies positive selection signals by searching for genomic regions that exhibit a sudden decline in haplotype richness. In total, we identified 14 positive selection signals, 11 using the classical approach and three using the HRiD approach, all together containing 34 annotated genes. The most reliable selection signal was mapped by all four approaches in the same region, overlapping between 13.17 and 13.60 Mb, and assigned to the CA5B, ZRSR2, AP1S2, and GRPR genes. High repeatability (86%) of results was observed, as 12 identified selection signals were also confirmed in other studies with sheep. HRiD offers an interesting possibility to be used complementary to other approaches or when only males are genotyped, which is often the case in genomic breeding value estimations. These results highlight the importance of the X-chromosome in the adaptive architecture of domestic ruminants, while our novel HRiD approach opens new possibilities for research.

## Introduction

The sheep (Ovis aries) was domesticated along with the goat and cattle around 11–12 kyBP on the Fertile Crescent from the wild Asian mouflon (Zeder, 2008). Today, more than 1,000 breeds of sheep are distributed in Asia (Lv et al., 2015), Europe (Ciani et al., 2020), Africa (Muigai and Hanotte, 2013), North America (Thorne et al., 2021), South America (McManus et al., 2010), and Australia (Nel et al., 2022), mainly because of their high multipurpose value as a source of milk, meat, wool, and fur (Lv et al., 2022), but also because of their high adaptability to different environments. Sheep are one of the most important livestock species in Croatia, found mainly in the coastal and mountainous regions along the East Adriatic and mostly kept extensively. Eight indigenous breeds (Cres Island Sheep, Dalmatian Pramenka, Dubrovnik Ruda, Istrian Sheep, Krk Island Sheep, Lika Pramenka, Pag Island Sheep, and Rab Island Sheep) form one East Adriatic sheep metapopulation (EAS) that constitutes the majority of sheep found in Croatia and is well representative of the Balkan sheep cluster. Recently, Ciani et al. (2020) showed that Balkan sheep breeds form a genetically specific cluster that is distinct from other European sheep breeds.

Despite the preference for dual milk and meat production, the genomic composition of EAS is largely modified according to environmental adaptation and sustainable production, since intensive artificial selection has never been practiced. Like many other native Mediterranean breeds (Ramón et al., 2021), EAS are characterised by high resilience to sudden climatic changes (e.g., sudden temperature changes, resistance to strong winds, and endurance to long periods of drought) and low nutrient requirements (ability to live on the barren karst pastures). In contrast to random stochastic changes (genetic drift), adaptation is usually a process characterised by systematic directional changes in gene frequencies at a few or numerous loci, ending with the fixation of favourable alleles and a decrease in variation at neighbouring loci. Consequently, these changes can be tracked on a genome as they are selection signals (footprints of adaptations) resulting from adaptation to the Mediterranean environment and production system.

Extreme Runs of Homozygosity Islands (eROHi), integrated Haplotype Score (iHS), and Number of Segregating Sites by Length (nSL) are three complementary and commonly used approaches to identify selection signals in livestock populations (Qanbari and Simianer, 2014; Utsunomiya et al., 2015; Saravanan et al., 2020). These within-population approaches rely on high-throughput analysis of genomic information from a single representative sample of a target population. Runs of homozygosity (ROH), a term coined by Lencz et al. (2007), are long homozygous regions in a genome that are thought to be autozygous because they are so long that it is unlikely that they are not descended from the same haplotype without being interrupted by recombination. With the emergence of empirical evidence on the genomic architecture of ROH, it has become clear that genomic regions with an extremely high frequency of SNPs in ROH (ROH islands according to Nothnagel et al., 2010) can be considered positive selection signatures (Boyko et al., 2010; Kim et al., 2013; Curik et al., 2014; Qanbari and Simianer, 2014). Here we use the term eROHi to refer to the statistical approach that identifies positive selection signals using ROH. Kardos et al. (2017) used computer simulations to show that complete and incomplete hard sweeps are more likely than soft sweeps to cause the frequency of ROH in genomic regions surrounded by the positively selected alleles. Today, the eROHi approach is widely used to identify selection signals in livestock populations (Gorssen et al., 2021). The integrated Haplotype Score (iHS) approach was developed by Voight et al. (2006) as an improvement to the Extended Haplotype Homozygosity (EHH) approach to reduce the influence of demographic history and is commonly used in diverse livestock species (Álvarez et al., 2020). The main idea of this approach is to compare the decay pattern of linkage disequilibrium of derived alleles (mutation) with the same pattern observed in ancestral alleles, implying neutrality (Qanbari and Simianer, 2014). According to Voight et al. (2006), iHS has the highest power in identifying intermediate selective sweeps or when the selected allele has an intermediate frequency that is not yet fixed. Number of Segregating Sites by Length (nSL) is another haplotype-based approach developed by Ferrer-Admetlla et al. (2014) that is able to detect soft and hard sweeps using genomic information from individuals in a single population. This approach is similar to iHS but is less sensitive to variations in recombination rate and has higher performance in detecting soft sweeps.

Compared to the numerous studies that have focused on identifying selection signals on autosomes, the same type of analysis, with the exception of a few comprehensive studies (Chen et al., 2018; Nadachowska-Brzyska et al., 2019), is severely underpowered on the sex chromosome (X or Z), leaving much room for a better understanding of selection behaviour on the sex chromosome as well as good potential for methodological improvements. The importance of this study is also supported by the particular characteristics of the X-chromosome compared to autosomes, such as genome size (∼5%), low mutation rate (0.015 mutations/Mb/generation), lower effective population size (3/4), lower recombination rates (2/3), and consequently higher linkage disequilibrium pointed out by Schaffner (2004). The occurrence of ploidy differences between hemizygous males, except for the small pseudo-autosomal region (PAR) that accounts for about 5% of the X-chromosome (Chen et al., 2018), and diploid females is the most important feature from the perspective of population genomics. For example, it is not possible to estimate genomic inbreeding and thus eROHi on a large portion of the X-chromosome (95%). In contrast, hemizygous status of males provides accurate haplotype information that can increase the accuracy of required phasing (Choi et al., 2018) in iHS and nSL approaches It has also been hypothesised that selection on X-chromosome genes is more efficient because all allele effects (including recessive alleles) are fully exposed to selection when expressed in hemizygous males (Vicoso and Charlesworth, 2006). Nevertheless, it is difficult to know whether we should expect a greater extent of selection signature compared to autosomes, because the strength of selection also depends on genetic drift, mutation and recombination rates, which are quite different on the X-chromosome. Certainly, further empirical analyses of selection patterns on the X-chromosome would improve our understanding of the joint effects of selection, genetic drift, mutation, and recombination rates.

The main objective of this study was to identify positive selection signals on the X-chromosome in EAS by three “classical” within-population approaches (eROHi, iHS, and nSL). In our analyses, all eight breeds representing the EAS were treated as a single metapopulation whose selection signals are similar and are mainly the consequence of a long-term adaptive response to the local (Mediterranean) environment and the applied production system. In addition, we proposed a new approach called Haplotype Richness Drop (HRiD) that uses information contained in male haplotypes. Our approach identifies positive selection signals by searching for genomic regions that exhibit a sudden decrease in allele (haplotype) richness and complements the other three methods used. For signals identified only by HRiD, we also performed phylogenetic network analysis of haplotypes in males to clarify their phylogenetic relationships (derived versus ancestral haplotype).

## Material and Methods

### Data, Genotyping and Quality Control

Our analyses were performed on the EAS represented by 202 individuals of eight native breeds sampled in Croatia: Cres Island Sheep (20), Dalmatian Pramenka (26), Dubrovnik Ruda (26), Istrian Sheep (25), Krk Island Sheep (20), Lika Pramenka (20), Pag Island Sheep (45) and Rab Island Sheep (20). To obtain a more representative sample and to exclude the influence of specific families on our results, EAS individuals were taken from 105 farms. In addition, we also sampled 10 mouflons from Rab island. All sampled sheep were raised in Croatia by registered breeders who provided information on their origin and the exact location of the farms. Sampling of close relatives (parents with offspring and full or half siblings) was avoided. Skin tissue samples from the ear were collected as part of the regular sampling of local autochthonous breeds by the National Gene Bank, from which DNA was isolated using a commercial kit (DNeasy Blood and Tissue Kits, Qiagene, Germany). Genotyping of 212 individuals was performed using the Ovine Infinium^®^ HD SNP BeadChip 600K (606 006 SNPs).

**FIGURE 1:**
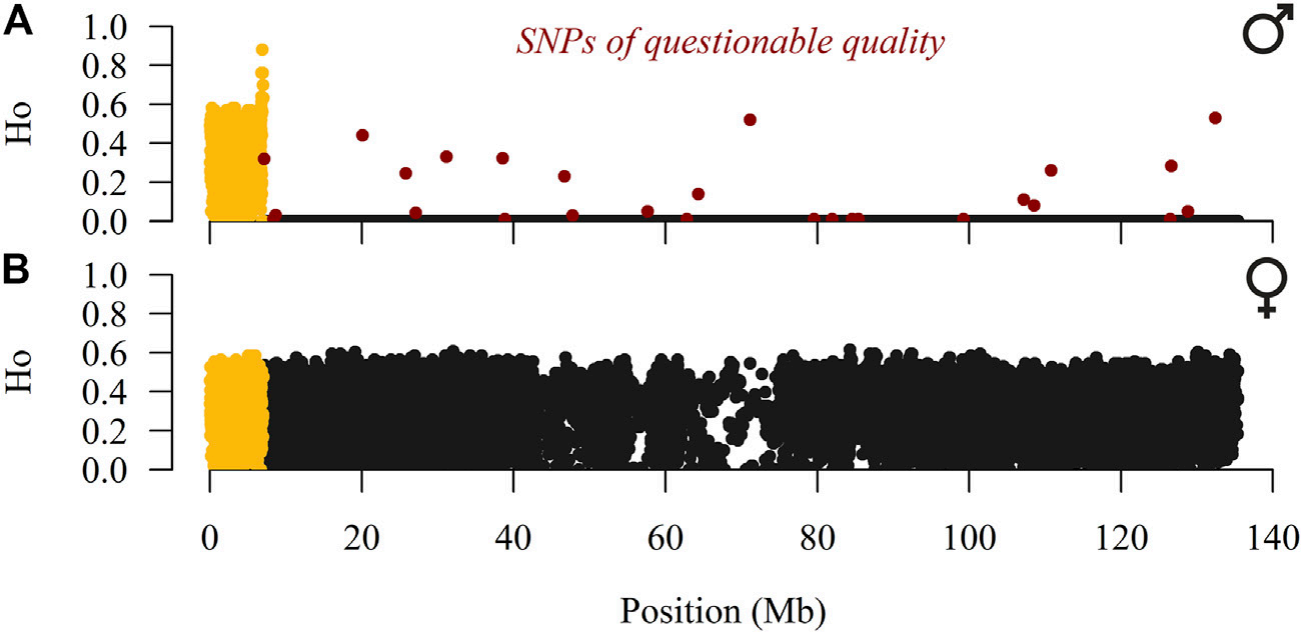
Observed heterozygosity (H_O_) of male **(A)** and female **(B)** individuals of SNPs placed over the X-chromosome (pseudo-autosomal SNPs are coloured in yellow) in the East Adriatic sheep metapopulation.

SAS 9.4. (SAS Institute, Cary, NC) and PLINK v1.9 software (Purcell et al., 2007; Chang et al., 2015) were used for quality control of genotypes. Our analyses started with 27,314 SNPs, all located on the X-chromosome according to the Oar v4.0 reference sheep genome. In addition, we excluded all SNPs with questionable quality (GenTrain score <0.4, GenCall score≤0.8, call rate <90%, and SNPs that deviated from the Hardy-Weinberg equilibrium with *p* < 10^–7^) and a Dalmatian Pramenka ram with call rate <95%. To localise the pseudo-autosomal region (PAR), the observed heterozygosity (H_O_) was calculated separately for males and females. Twenty-seven heterozygous SNPs placed in the hemizygous part of the X-chromosome were considered mis-genotyped (SNPs highlighted in red in Figure 1) and excluded from further analyses. For the first 1232 SNPs (0.03–7.03 Mb), males had an average H_O_ = 0.34, whereas almost all remaining SNPs (17751; 7.04–135.40 Mb) had H_O_ = 0. In contrast, females had an average H_O_ = 0.32 for the first 1232 SNPs and across the entire X-chromosome. Therefore, the PAR likely spans from 0.00 to 7.04 Mb, which is consistent with the mapped PAR (0.00–7.05 Mb) in the reference sheep genome (Jiang et al., 2014). We continued work on a baseline dataset of 18,983 SNPs (1,232 SNPs were placed on the pseudo-autosomal portion) genotyped in 201 sheep individuals (100 males and 101 females) and 10 mouflons, whose genotypes were used to evaluate ancestral and derived alleles and to construct the phylogenetic relationship. The maximum and mean distances between adjacent SNPs were 232 and 7.13 kb, respectively.

In this study, additional attention was paid to the mode of selection and the best use of all available genotyping information. Therefore, we applied three complementary approaches (eROHi, iHS, and nSL), all related to haplotype-based statistics and commonly used to identify soft and hard signals of positive selection (soft and hard selective sweeps) within populations. The use of different methods also allowed more efficient use of available genotyping information. For example, in the iHS and nSL approach, we used the entire information of 201 available genotypes (male and female), whereas the eROHi approach was performed with only 101 female genotypes. Here, we also proposed a new approach called HRiD based only on the male haplotypes (100 rams). The information on the most frequent allele in the mouflons was used to define the ancestral information (ancestral versus derived mutation), which is the preferred option in the iHS and nSL approach.

### Extreme Runs of Homozygosity Islands

Prominent SNPs that occurred with high frequency in ROHs in a population of 101 females were considered indicators of genomic regions subject to positive selection. Only segments with 15 or more consecutive homozygous SNPs, with a maximum distance of 250 Kb between two SNPs, and a density of at least one SNP per 20 Kb were considered ROHs. The mean and maximum distance between adjacent SNPs were considered when determining the values for the previous parameters. The minimum ROH length was set at 0.25 Mb, which is an extremely low value compared to other studies. This was done intentionally based on SNP coverage analysis, as on average 35 SNPs covered 0.25 Mb, while the maximum distance and minimum density excluded low-density regions. If the eROHi approach is based on ROH > 1 Mb, we are looking for ROHs that originated in the last 50 generations. Because we were particularly interested in identifying signals reflecting a long-term adaptive response, we allowed calculation of much shorter ROHs (>0.25 Mb) to more precisely track the selection pattern that arose in the last 200 generations. ROHs were estimated using the SNP & Variation Suite (SVS) v8.7.0 software package (Golden Helix, Inc., Bozeman, MT, www.goldenhelix.com). ROH were calculated separately for each of the six classes with different lengths (0.25–1.00, 1.00–2.00, 2.00–4.00, 4.00–8.00, 8.00–16.00, and >16.00 Mb) to account for the genotyping error rate of the HD SNP chip (0.25%). The number of allowed heterozygotes and missing SNPs in all classes above 1 Mb was defined according to Ferenčaković et al. (2013), while no heterozygotes or missing SNPs were allowed in the 0.25–1.00 Mb class. Subsequently, the ROH frequency was calculated for each SNP and then normalised by the mean frequency, while the transformed value was represented as −log(P). SNPs with −log(P) ≥ 3.3 were considered outliers, whereas chromosomal regions with consecutive outliers were considered significant. The significance threshold [−log(P) ≥ 3.3] corresponded to a frequency of 0.396 (40 individuals) and was calculated using the simpleM method (Gao et al., 2008). At the end of the analysis, signals are ranked according to the highest −log(P) value within a signal (“peak of signal”).

### Integrated Haplotype Score and Number of Segregating Sites by Length

iHS (Voight et al., 2006) and nSL (Ferrer-Admetlla et al., 2014) are closely related methods based on haplotype homozygosity of ancestral and derived alleles at each core SNP and are used to detect positive selection signals from soft sweeps. However, the main difference is that nSL measures haplotype length based on the number of segregating sites rather than actual genomic distance. Consequently, no genetic map is required to calculate the statistic, and robustness to variation in recombination and/or mutation rate is increased (Ferrer-Admetlla et al., 2014).

Haplotype phasing required for iHS and nSL was performed using Shapeit2 software (Delaneau and Marchini, 2014), with the “--chrX” option that allowed the use of information from male and female genotypes (201). The VCF file was recoded so that the ancestral allele was the reference and the derived allele was the alternative (R script provided in Supplementary File S1). Subsequently, iHS values for each SNP were calculated using the R package “rehh” (Gautier and Vitalis, 2012), whereas nSL values were calculated using the software selscan (Szpiech and Hernandez, 2014) without constraints (the allowed values for gap-scale and max-gap were higher than the maximum distance in our data set).

All iHS and nSL values were normalised within the frequency bin size of 0.025, and −log(P) values were calculated assuming two-sided tests because both extremely positive and extremely negative iHS or nSL values were considered informative (derived and ancestral alleles). According to Voight et al. (2006), it is more informative to look for windows of consecutive SNPs that contain numerous extreme values because selective sweeps tend to generate clusters of extreme values in the sweep region, whereas in a neutral model the extreme values are more evenly distributed. Thus, we used the sliding window approach (500 kb size; 100 kb slide) and considered SNPs with −log(P) > 2 as outliers and nonoverlapping windows with >10% outliers as significant signals (ordered by their proportion). Furthermore, because both methods are ratio-based, they are limited in their ability to detect sweeps that are very close to fixation, so for any SNP with a minor allele frequency <5%, a calculation was not possible. Therefore, they were assigned the value NA, but they were retained for the construction of haplotypes in adjacent SNPs and are also informative for the eROHi and HRiD approaches.

### Haplotype Richness Drop: Explanation and Derivation of the Concept

With the idea of maximising the use of all available genotyping information, we propose a new approach that uses the information contained in male haplotypes to identify genomic regions that exhibit positive selection signals. The proposed method is based on the calculation of the effective number of alleles, defined and interpreted by Kimura and Crow (1964) “as the expected value of the sum of squares of the allele frequencies, or more simply as the reciprocal of the effective number of alleles maintained in the population.” In conservation genetics, the effective number of alleles is considered a measure of allelic richness and is defined by the symbol A_e_ or n_a_ (Allendorf et al., 2013; Greenbaum et al., 2014). On the X-chromosome without PAR, male genotypes are hemizygous, making it easy to derive exact haplotypes of different lengths. For this reason, the term allele has been replaced and we continue to use the term effective number of haplotypes here as a measure of haplotype richness (n_h_).

Our main assumption is that the presence of positive selection leads to a sudden decrease in the effective number of haplotypes, which was measured by calculating Haplotype Richness Drop values (HRiD) defined by the following formula;

$$HRiD_{{\text{w}}_{\text{i}+1}}=\frac{n_{h_{{\text{w}}_{\text{i}}}}+\;n_{h_{{\text{w}}_{\text{i}+2}}}}{2n_{h_{{\text{w}}_{\text{i}+1}}}}$$

where $n_{h_{{\text{w}}_{\text{i}}}}$ represents the effective number of haplotypes of the i^th^ sliding window (haplotype) under study (*i* = 1, … ,w_i_, where w_i_ = 503 and w_i+2_ = 505). For the first (w_1_) and last window (w_i+2_), the formula of the numerator is slightly different to allow identification of selection signals in these two windows. Thus, the numerator for the first window $2n_{h_{{\text{w}}_{2}}}$ , while the numerator for the last window is $2n_{h_{{\text{w}}_{\text{i}+1}}}$ . Note that the effective number of haplotypes for the haplotypes defined in each window is calculated as the reciprocal of the sum of their squared haplotype frequencies, whereas the R script that enables the calculation of HRiD can be found in Supplementary File S2. If there is no selection, HRiD values should fluctuate around the value of one, whereas positive selective sweeps would lead to higher positive values because n_h_ is much lower compared to surrounding regions. With this approach, the selection signals detected by HRiD do not depend on the heterogeneity of recombination rates. The size of the window was set to 70 SNPs with a slider of 35 SNPs (average ≈ 500 Kb and 250 Kb) to allow direct comparison of signals with those obtained by other methods. HRiD approach is expected to detect efficiently signals of positive selection that resemble hard sweeps. HRiD values were normalized and converted to −log(P) values. Windows (haplotypes) with −log(P)≥3.3 corresponded to a minimum HRiD value of 2.8 and were considered significant.

To illustrate the phylogenetic relationship between ancestral and derived haplotypes in the selection signals obtained by HRiD and classical approaches, we created a number of Median-joining networks (MJN). Our phylogenetic analysis was based on 100 male sheep and five male mouflon, which we assumed to represent the ancestral haplotypes. First, the SNPs of the X-chromosome were converted to fasta format using the R package seqRLFP (Ding and Zhang, 2012) and visualised using MEGA7 (Kumar et al., 2016). In addition, we used DnaSP (Rozas et al., 2017) to derive unique haplotypes for each selection signal identified, except for two signals that were merged due to overlap, while MJNs (Bandelt et al., 1999) were generated using both Arlequin v. 3.5.2.2 (Excoffier and Lischer, 2010) and PopART software (Leigh and Bryant, 2015).

### Gene Annotation and Functional Characterization of Candidate Regions

Annotation of genes was performed within significant signals of positive selection using information from the SNP & Variation Suite (SVS) v8.7.0 software package (Golden Helix, Inc., Bozeman, MT, www.goldenhelix.com) based on positions in the OAR4.0 reference sheep genome. Functional analysis of candidate genes was performed using the UniProt (https://www.uniprot.org/) and GeneCards (https://www.genecards.org) platforms. In addition to genomic information from sheep (UniProt), genomic information from other species, including humans (GeneCards) and cattle (UniProt), was also used.

## Results

### Signals of Positive Selection Mapped by eROHi, iHS, and nSL

**FIGURE 2:**
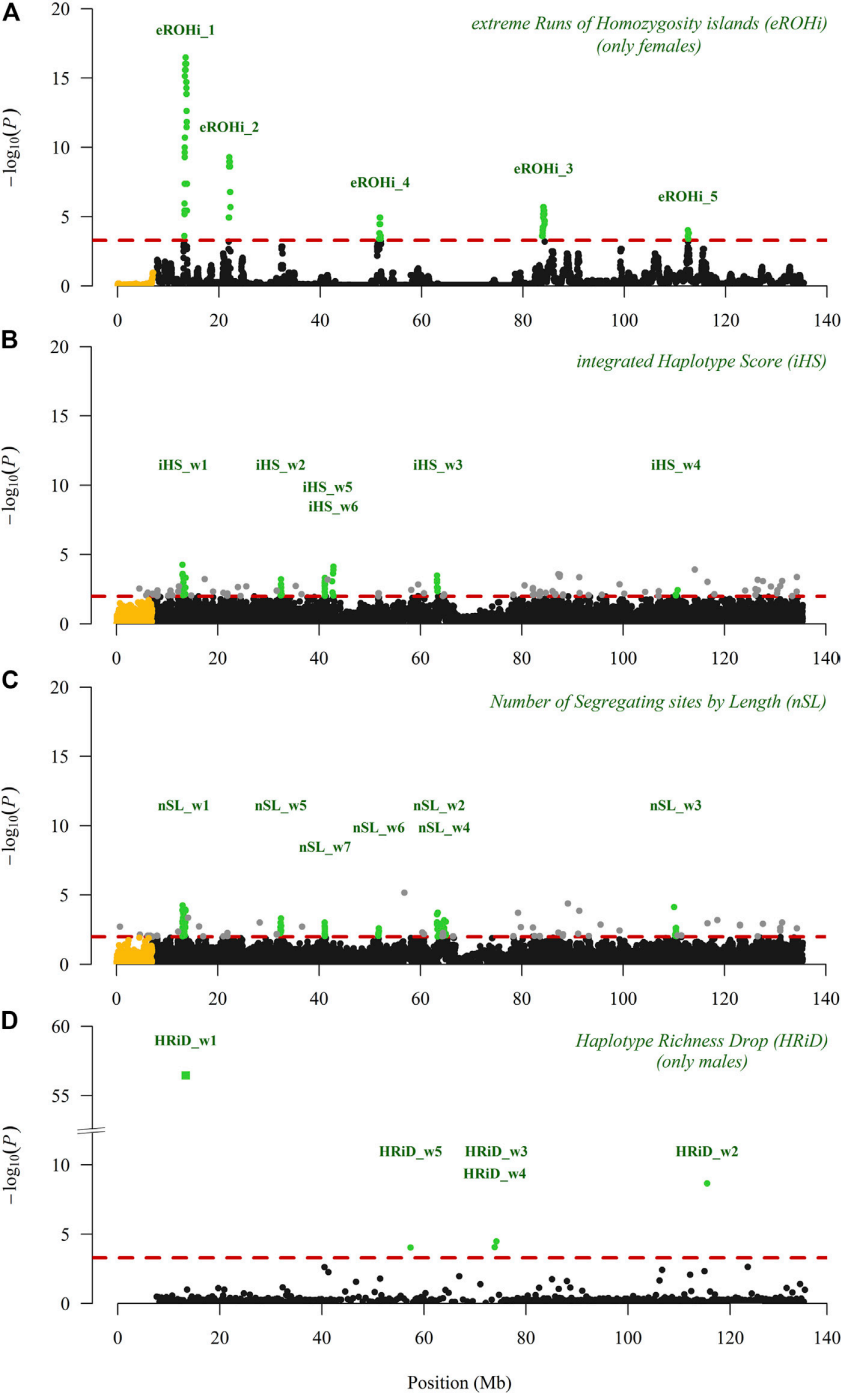
Visualisation of positive selection signals in the Manhattan plot analysed on the X-chromosome (pseudo-autosomal SNPs are coloured in yellow) in East Adriatic sheep using three “classical” (eROHi, iHS and nSL) and one new (HRiD) approach; **(A)** eROHi, **(B)** iHS, **(C)** nSL, and **(D)** HRiD. SNPs or windows above the dashed threshold line (in red) that were considered significant are coloured green, except for single SNP outliers (grey) observed in the iHS and nSL approaches.

Figures 2A–C shows the visualisation of positive selection signals in the Manhattan plot analysed with three “classical” approaches. In our analyses performed with eROHi, iHS, and nSL, we detected nine genomic regions with 11 positive selection signals. We are quite confident that the genomic region mapped from 13.10 to 13.69 Mb correctly indicated the positive selection signals, as it was identified by all three approaches used. At the same time, this selection signal had the highest -log(P) value with eROHi (16.5) and the highest proportion of outliers with iHS (17/50) and nSL (35/50). The results of iHS and nSL were very similar, as five selection signals (from 13.10 to 13.60 Mb, from 32.20 to 32.80 Mb, from 41.00 to 41.50 Mb, from 63.20 to 63.80 Mb, and from 110.10 to 110.80 Mb) were identified by both approaches (Figures 2B,C). However, some other signals were identified only by iHS (from 42.50 to 43.00 Mb) or by nSL (from 51.40 to 51.90; from 63.80 to 64.30 Mb; and from 64.60 to 65.10 Mb). One selection signal identified by eROHi (eROHi_4), ranging from 51.63 to 51.94 Mb (Figure 2A), was also identified by the nSL approach (nSL_w6) (Figure 2C), whereas three selection signals (ranging from 21.96 to 22.26 Mb, from 83.78 to 84.28 Mb, and from 112.53 to 112.72 Mb) were identified only by eROHi (Figure 2A).

### Signals of Positive Selection Mapped by New HRiD Approach With Phylogenetic Analysis

In Figure 2D, we visualised the positive selection signals in the Manhattan plot analysed with our new HRiD approach. HRiD was able to identify four (five if we split the signal located from 73.57 to 74.54 into two signals) genomic regions showing positive selection signature patterns. The largest selection signal observed by HRiD with −log(P) equal to 56.5 was located from 13.04 to 13.62 (HRiD_w1) and largely overlapped with the other three “classical” approaches (eROHi, iHS, and nSL).

This result confirms both the reliability of HRiD in identifying selection signals and our confidence in this identified positive selection signal. All other selection signals identified by HRiD (from 56.64 to 58.09 Mb, from 73.57 to 74.20 Mb, from 73.90 to 74.54 Mb, and from 115.30 to 115.73 Mb) were not confirmed by other approaches. As a consequence of our methodological approach, the definition of the window size used in HRiD, the selection signals from 73.57 to 74.20 Mb and from 73.90 to 74.54 Mb were reported as two signals, although it would be more appropriate to consider them as one large signal from 73.57 to 74.54. Part of this signal (HRiD_w4) had the smallest n_h_ value (1.9), which could be influenced by the low recombination rate. More detailed information about the significance level of the identified selection signals can be found in Tables 1, 2.

**TABLE 1:** Description of mapping statistics and annotation of genes in selection signals on the X-chromosome in East Adriatic sheep by three classical (eROHi, iHS and nSL) approaches.

Signal Name	Position (Mb)	SNPs*	−log(P)^$^	Candidate Genes under selection^#^
eROHi_1	13.17–13.69	59/59	16.5	***CA5B*** *,* ***ZRSR2*** *,* ***AP1S2*** *,* ***GRPR***
eROHi_2	21.96–22.26	35/35	9.3	*POLA1, ARX*
eROHi_3	83.78–84.28	73/73	5.7	No annotated genes found
eROHi_4	51.63–51.94	33/33	4.9	***DGKK*** *,* ***CCNB3***
eROHi_5	112.53–112.72	11/11	4.0	*PLS3*
iHS_w1	13.10–13.60	17/50	4.3	***TMEM27*** *,* ***CDC42*** *,* ***CA5B*** *,* ***ZRSR2*** *,* ***AP1S2*** *,* ***GRPR***
iHS_w2	32.20–32.70	13/55	3.2	No annotated genes found
iHS_w3	63.20–63.70	6/35	3.5	*RLIM,* ***KIAA 2022*** *,* ***ABCB7***
iHS_w4	110.30–110.80	5/36	2.4	***DOCK11*** *, WDR44, KLHL13*
iHS_w5	41.00–41.50	9/65	3.3	***NDP*** *,* ***EFHC2***
iHS_w6	42.50–43.00	6/60	4.1	*MIR221*
nSL_w1	13.10–13.60	35/50	4.3	***TMEM27*** *,* ***CDC42*** *,* ***CA5B*** *,* ***ZRSR2*** *,* ***AP1S2*** *,* ***GRPR***
nSL_w2	63.30–64.30	19/44	3.7	***KIAA 2022*** *,* ***ABCB7*** *, UPRT, ZDHHC15, MAGEE2*
nSL_w3	110.10–110.60	12/39	4.1	***DOCK11***
nSL_w4	64.60–65.10	10/39	3.2	*MAGT1, ATRX, FGF16*
nSL_w5	32.30–32.80	14/61	3.3	No annotated genes found
nSL_w6	51.40–51.90	8/56	2.6	*SHROOM4,* ***DGKK*** *,* ***CCNB3***
nSL_w7	41.00–41.50	9/65	3.0	***NDP*** *,* ***EFHC2***

*Number of significant/all SNPs within the signal (window): −log(P) = 3.3 for eROHi,−log(P) ≥ 2 for iHS and nSL approach. ^$^The highest −log(P) value for the individual SNP within the signal (window). ^#^Genes identified with at least two approaches (eROHI, iHS or nSL) as positive selection signals are bolded.

**TABLE 2:** Description of mapping statistics and annotation of genes in selection signals on the X-chromosome in East Adriatic sheep by new HRiD approach.

Signal Name	Position (Mb)	n_a_*	n_h_^$^	HRiD	−log(P)^#^	Candidate Genes under selection^*‡*^
HRiD_w1	13.04–13.62	42	5.4	9.6	56.5	***TMEM27*** *,* ***CDC42*** *,* ***CA5B*** *,* ***ZRSR2*** *,* ***AP1S2*** *,* ***GRPR***
HRiD_w2	115.30–115.73	36	13.3	4.2	8.7	*AMOT, LHFPL1*
HRiD_w3	73.90–74.54	13	4.3	3.2	4.5	*DACH2*
HRiD_w4	73.57–74.20	10	1.9	3.1	4.1	*CHM, DACH2*
HRiD_w5	56.64–58.09	33	6.9	3.1	4.0	*AR, OPHN1, YIPF6*

*Total number of unique alleles (haplotypes). ^$^Effective number of alleles (haplotypes), Haplotype Richness Drop score (HRiD). ^#^−log(P) value refers to the significance of the signal (window). ^‡^Genes additionally identified as positive selection signals by other approaches (eROHI, iHS or nSL) are bolded.

**FIGURE 3:**
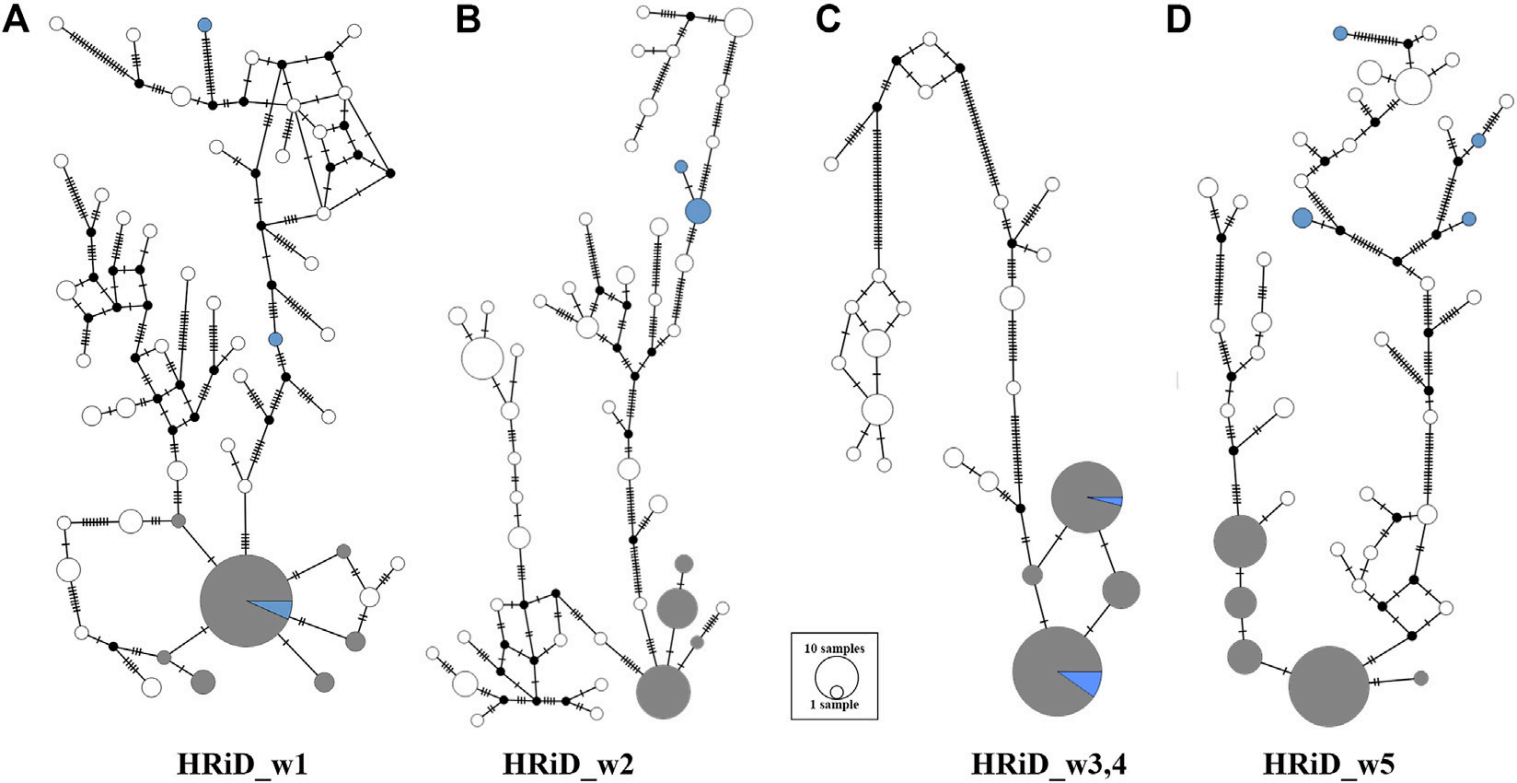
Median-joining network showing the phylogenetic relationship between ancestral and derived haplotypes representing mapped selection signals identified by HRiD; **(A)** HRiD_w1 (70 SNPs from 13.04 to 13.62 Mb), **(B)** HRiD_w2 (70 SNPs from 115.30 to 115.73), **(C)** HRiD_w3 and HRiD_w4 (105 SNPs from 73.57 to 74.54), and **(D)** HRiD_w5 (70 SNPs from 56.64 to 58.09). The most common haplotypes with adjacent haplotypes no more than three mutations apart are coloured grey, whereas the mouflon haplotypes (representing ancestral haplotypes) are coloured light blue.

Another good feature of HRiD is that it allows further phylogenetic analysis of haplotypes that show positive selection signals. Here, we analysed the phylogenetic relationship (MJN) between all haplotypes defined by the HRiD approach (Figure 3). Five male mouflons were also included in the analyses because we assumed that their most common haplotypes had an ancestral origin. MJN was performed only for the four selection signals because two signals (HRiD_w3 and HRiD_w4) were considered as one signal. For this reason, the haplotypes located within this signal were longer (105 SNPs) than the haplotypes (70 SNPs) located within the other three selection signals. The most common haplotype is likely to be the most favourable haplotype selected together with the neighbouring haplotypes (here we assumed that they are not more than three mutations away). Following this concept, the most common mouflon haplotype for the selection signal HRiD_w1 is in the group of favourable haplotypes, indicating that the ancestral haplotype is subject to positive selection (Figure 3A). The same pattern, namely that the ancestral haplotype is subject to positive selection, was observed for the merged selection signal HRiD_w3,4 (HRiD_w3 and HRiD_w4) (see Figure 3C). In contrast, favourable haplotypes under positive selection in signals HRiD_w2 and HRiD_w5 were considered derived because they were far from the ancestral haplotypes present in the mouflons (Figures 3B,D).

### Gene Annotation and Functional Characterization of the Identified Signals

The description of mapping statistics and annotation of genes for the identified selection signals on the X-chromosome in EAS by three “classical” and our new approach are shown in Tables 1, 2, respectively. A total of 34 genes in 12 identified regions were found to have patterns of selection signatures. No annotated genes were found in two genomic regions that showed patterns of positive selection signals. The region from 32.20 to 32.80 was identified by iHS and nSL, whereas the region from 83.78 to 84.28 was identified only by the eROHi approach.

#### Candidate Genes Assigned to the Selection Signal Between 13.04 and 13.69 Mb

Four genes (*CA5B*, *ZRSR2, AP1S2*, and *GRPR*) were within the main signal of all four approaches and can be considered as major candidates, ahead of *TMEM27* and *CDC42*, which were not identified using only the eROHi approach. *CA5B* (Carbonic Anhydrase 5B) expression is localized in mitochondria and involved in biological functions such as reversible hydration of carbon dioxide and response to bacteria. In addition, *CA5B* may play an important role in growth, development, energy storage and utilization of porcine skeletal muscle (Guo et al., 2021). *ZRSR2* (Zinc Finger CCCH-Type, RNA binding motif and Serine/Arginine Rich 2) may play a role in network interactions during spliceosome assembly and has been linked to sex determination in cattle (Peterson, 2020). *AP1S2* (Adaptor Related Protein Complex 1 Subunit Sigma 2) has been linked to abnormal responses to novelty, while *GRPR* (Gastrin Releasing Peptide Receptor) regulates multiple functions of the gastrointestinal tract and central nervous system and has been linked to regulation of the reproductive system in boars (Ma et al., 2018). *TMEM27* (Collectrin) is important for amino acid transport, while *CDC42* (Cell Division Cycle 42) regulates signalling pathways that control various cellular functions such as cell morphology, migration, endocytosis, and cell cycle progression.

#### Candidate Genes Assigned to the Selection Signal Between 21.96 and 22.26 Mb

*POLA1* (DNA Polymerase Alpha 1, Catalytic Subunit) and *ARX* (Aristaless Related Homeobox) genes were mapped by eROHi. *POLA1* gene was associated with DNA replication and RNA primer synthesis. In addition, Starokadomskyy et al. (2021) linked it to growth, intellectual abilities, and immune disorders, while *ARX* is thought to be involved in CNS development.

#### Candidate Genes Assigned to the Two Selection Signals Between 41.00 and 43.00 Mb

The *NDP* and *EFHC2* genes were found to range from 41.00 to 41.50 Mb. *NDP* (Norrin Cystine Knot Growth Factor NDP) encodes a secreted protein with a cysteine knot motif that activates the Wnt/beta-catenin signalling pathway and has been associated with dysplasia in dogs (Joyce et al., 2021), whereas *EFHC2* (EF-Hand Domain Containing 2) is associated with fear recognition and harm avoidance in humans (Blaya et al., 2009). The iHS signal mapped from 42.50 to 43.00 Mb contains only the gene *MIR221* (MicroRNA 221), which has been associated with the regulation of milk fat, protein synthesis, and mammary gland development in sheep (Duman et al., 2021).

#### Candidate Genes Assigned to the Selection Signal Between 51.40 and 51.94 Mb

*DGKK* and *CCNB3* genes mapped by eROHi and nSL, whereas S*HROOM4* was mapped only by nSL. *DGKK* (Diacylglycerol Kinase Kappa) is involved in oxidative stress response, while *CCNB3* (Cyclin B3) plays an essential role in cell cycle control and was dispensable for spermatogenesis in mice (Karasu and Keeney, 2019). In addition, *SHROOM* (Shroom Family Member 4) plays an important role in regulating cytoskeletal architecture, brain development, and cognition.

#### Candidate Genes Assigned to the Selection Signal Between 56.64 and 58.09 Mb

Three genes (*AR, OPHN1* and *YIPF6*) mapped by HRiD only are associated with tail fatness in sheep (Moradi et al., 2012). The gene *AR* (Androgen Receptor) is also important for prostate development, urogenital system, and reproduction and has been linked to carcass traits in cattle by Choi et al. (2010). *OPHN1* (Oligophrenin 1) has been linked to abnormal response to novelty, while *YIPF6* (Yip1 Domain Family Member 6) has been linked to intestinal epithelial cell development.

#### Candidate Genes Assigned to the Two Selection Signals Between 63.20 and 65.10 Mb

The following genes were found in the remaining common iHS/nSL selection signals. Thus, the *RLIM*, *KIAA2022* and *ABCB7* genes were annotated in the signal from 63.20 to 63.80 Mb. *RLIM* (Ring Finger Protein, LIM domain interacting) was associated with ligase activity and transcriptional corepressor activity. It has also been linked to mouse lung development (Kammoun et al., 2018) and spermiogenesis (Wang et al., 2021). *KIAA 2022* (Neurite Extension And Migration Factor) has been linked to nervous system development, while *ABCB7* (ATP Binding Cassette Subfamily B Member 7) is involved in the transport of heme from mitochondria to the cytosol and has therefore been linked to mitochondrial iron accumulation. Three genes found by nSL and mapped in the signal from 63.80 to 64.30 Mb (*UPRT*, *ZDHHC15* and *MAGEE2*). *UPRT* (Uracil Phosphoribosyltransferase homolog) was associated with nucleoside metabolic process, lactation and female pregnancy, while *MAGEE2* (MAGE Family Member E2) may play a role as a tumor antigen. The following signal mapped from 64.60 to 65.10 Mb by nSL contained *MAGT1*, *ATRX* and *FGF16* genes. *MAGT1* (Magnesium Transporter 1) is associated with the immune system and glycosylation (Blommaert et al., 2019), while *ATRX* (ATRX chromatin remodeler) has numerous functions in development. *FGF16* (Fibroblast Growth Factor 16) is associated with embryonic development, cell growth, morphogenesis, tissue repair, tumor growth, and proper heart development.

#### Candidate Genes Assigned to the Selection Signal Between 73.57 and 74.54 Mb

Two genes (*CHM* and *DACH2*) were mapped by HRiD only. Annotations associated with *CHM* (CHM Rab Escort Protein) include GTPase activator activity and Rab geranylgeranyltransferase activity and have been linked to milk production in cattle (Stella et al., 2010). *DACH2* (Dachshund Family Transcription Factor 2) may be involved in the regulation of organogenesis and myogenesis and may play a role in premature ovarian failure.

#### Candidate Genes Assigned to the Selection Signal Between 110.10 and 110.80 Mb

Three genes (*DOCK11*, *WDR44* and *KLHL13*) were mapped by iHS, whereas the *DOCK11* gene was mapped by nSL only. *DOCK11* (Dedicator Of Cytokinesis 11) is involved in the polarisation processes of epithelial cells. Annotations of this gene include guanyl nucleotide exchange factor activity and binding of small GTPases. *WDR44* (WD Repeat Domain 44) may be involved in vesicle recycling, while *KLHL13* (Kelch Like Family Member 13) is required for proper chromosome segregation and completion of cytokinesis and underlies the female pluripotency phenotype in mammals (Genolet et al., 2021).

#### Candidate Genes Assigned to the Selection Signal Between 112.53 and 112.72 Mb

*PLS3* (Plastin 3) was the only gene found by eROHi in this region. It is related to the binding of calcium ions and actin and may play a role in regulating bone development.

#### Candidate Genes Assigned to the Selection Signal Between 115.30 and 115.73 Mb

Two genes, *AMOT* (Angiomotin) and *LHFPL1* (LHFPL tetraspan subfamily member 1), were mapped to this region only by HRiD. *AMOT* has been linked to convergent evolution and domesticated adaptation to high-altitude environments in humans (Witt and Huerta-Sánchez, 2019) and, together with *LHFPL1*, to hypoxia adaptation in dogs (Wu et al., 2016).

## Discussion

We performed fine mapping of positive selection signals of the X-chromosome in EAS using three classical (eROHi, iHS, and nSL) and one new (HRiD) approach used here for the first time. All selection mapping approaches used in this study are classified as intra-population analyses. In this way, we chose analyses in which the results are not affected by the choice of breeds to be compared, which may be the case for inter populations analyses (Saravanan et al., 2020). Our analyses were based on 201 sheep (101 females and 100 males) and 10 mouflon animals (five females and males) genotyped with the high-density array. The X-chromosome was covered by 18,983 SNPs, with a mean distance between adjacent SNPs of 7.13 kb. This high-density information increased the accuracy of our results. For example, in estimating eROHi, we were able to detect ROHs as short as 0.25 Mb and analyse selection signals over an extended period of time (200 generations). Álvarez et al. (2020) also tracked selection signals on a longer time scale, but their analyses were based on estimation of homozygosity-by-descent (HBD) segments. For more information on the HBD approach, see Druet and Gautier (2017) and Solé et al. (2017). We chose the eROHi approach because we assumed that the ROH approach evaluates autozygosity caused by both inbreeding and selection (Curik et al., 2002), whereas the HBD approach focuses more on deviations from HWE caused by inbreeding rather than selection (Druet and Gautier, 2017). However, our assumption that the eROHi approach better captures selection-induced autozygosity remains to be verified by computer simulations.

The use of all information available to us was maximised so that eROHi was performed only on female genotypes, iHS and nSL were performed on all genotypes (optimal use of the “--chrX” option in the Shapeit2 software), whereas HRiD was performed only on male haplotypes (SNPs outside PAR). We are not aware of any other study in which mapping of positive selection on the sex chromosome was performed only on male (XY) or female (XZ) genomic information. From this point of view, HRiD offers an interesting possibility to be used complementary to other approaches or when only male genomic information is available, which is often the case in genomic breeding value estimations.

In total, we identified 12 regions on a 135.4 Mb long, covered by SNP array, sheep X-chromosome in EAS that have genomic patterns characteristic of the selection signatures (14 signals). While 11 selection signals were identified using three “classical” approaches (eROHi, iHS and nSL), three additional signals were identified using HRiD, our novel approach for detecting selection signals from haplotype information of male individuals (not PAR). In the 12 identified regions, we annotated 34 genes, as two regions (from 32.20 to 32.80 Mb and from 83.78 to 84.28 Mb) had no genes annotated.

Almost all, 11 of 14, selection signals identified in this study were also obtained (overlapping intervals) in similar studies conducted on domestic or wild sheep populations, although the signal intervals did not completely overlap (Supplementary Table S1). The exceptions were selection signals mapped from 21.96 to 22.26 and from 83.78 to 84.28 by eROHi, and a selection signal mapped from 115.30 to 115.73 Mb by HRiD, all of which were not mentioned in other sheep studies. Thus, although the 115.30–115.73 Mb region has not been detected in other studies with sheep, functional characterization of the corresponding genes (*AMOT* and *LHFPL1*) suggests that they may be candidates for adaptation (hypoxia, more details in Results section). Our most reliable signal overlapped with the region mapped from 13.20 to 13.60 Mb (annotated for the *CA5B*, *ZRSR2*, *AP1S2* and *GRPR* genes) by Chen et al. (2018) in a large study of 68 sheep breeds worldwide (iHS) and in a comparison between sheep and mouflon (XP-EHH). In both approaches, part of this region (13.2–13.4 Mb; *CA5B*, *ZRSR2*, *AP1S2*) was classified as the first signal, indicating its importance as well as the biological function of the annotated genes. The mapped region (iHS and nSL) from 32.20 to 32.80 Mb, without annotated genes, was also mapped by Zhu et al. (2015), Liu et al. (2016) and Chen et al. (2018). Similarly, the mapped region (iHS and nSL) from 41.00 to 43.00 Mb, with *NDP* and *EHC2* genes, was confirmed by Zhu et al. (2015); Liu et al. (2016) and Cesarani et al. (2022). Since EFHC2 is associated with fear recognition and harm avoidance, we can assume a connection of this signal with extensive husbandry (fear of guard dogs and wolves), which is characteristic of Croatian indigenous breeds. The mapped region (eROHi and nSL) from 51.40 to 51.94 was identified in two studies by Zhu et al. (2015); Zhu et al. (2020). The very large signal mapped here from 56.64 to 58.09 by HRiD and containing three (*AR*, *OPHN1* and *YIPF6*) annotated genes was found in four other studies (Liu et al., 2016; Chen et al., 2018; Manzari et al., 2019; Cesarani et al., 2022). This concordance was of interest to us because this region was not detected by eROHi, iHS, and nSL in this study but coincided with the most significant individual nSL outlier [−log(P) = 5.17)] at position 56.81 Mb, demonstrating the complementary potential of HRiD to identify positive selection signals. The signal from 64.60 to 65.10 Mb (*MAGT1*, *ATRX* and *FGF16*) was mapped only by nSL and identified by Chen et al. (2018) and Zhu et al. (2020) in domestic sheep, but also by Kardos et al. (2015) for the candidate genes *ATRX* and *FGF16* in bighorn sheep. Another selection signal mapped in this study using HRiD but not classical approaches relates to the region from 73.57 to 74.54 (annotated for the genes *CHM* and *DACH2*) and was also identified by Zhu et al. (2015); Zhu et al. (2020), further highlighting the usefulness of the HRiD approach. The signal assigned to (iHS and nSL) the region from 110.10 to 110.80 (*DOCK11*, *WDR44* and *KLHL13*) was also identified as a positive selection signal by Chen et al. (2018), while the signal assigned to (eROHi) the region from 112.53 to 112.72 (*PLS3*) was also assigned by Zhu et al. (2015).

By analysing the phylogenetic relationship (MJN) between all haplotypes within each signal defined by the HRiD approach, we were able to determine whether the ancestral or derived haplotype was subject to selection. Because mouflons (ancestral haplotype) are well adapted to their natural environment (no artificial selection occurred), we hypothesized that signals from the ancestral haplotype were adaptation-related candidate regions (HRiD_w1 and HRiD_w3,4).

In addition to mapping positive selection of the X-chromosome in EAS, this study also has a methodological component related to the explanation of the HRiD approach. HRiD results might be sensitive to the definition of window size, but this is a feature of other approaches, as age and strength of selection are functionally related to haplotype size. In comparison to some other approaches, HRiD has some positive aspects, such as lower sensitivity to variation in recombination rate and the possibility of phylogenetic analyses of haplotypes observed in genomic regions that exhibit patterns of selection signatures. Overall, we believe that the basic idea of the HRiD approach is sound, while there is room for additional improvements that we hope to achieve in the near future. It is important to note that the most important and difficult parameter in the HRiD approach is the size of the window to be determined. Since the SNP array density varies from region to region, we suggest setting the size based on the number of SNPs rather than bp units so that the calculated n_h_ values are more representative (determined with the same number of SNPs). Moreover, as in the other approaches (iHS and nSL), the size should be set according to the population studied. We set the number of SNPs to 70 (35), which corresponds to an average of 500 kb (250 kb) and is consistent with other approaches. Because the genomic composition of EAS has changed significantly due to environmental adaptations and sustainable production, we focused on signals that have been present in the population for a long time (approximately 100–200 generations). HRiD is less efficient for selection signals longer than the defined window size because adjacent windows are also subject to selection. This may have been the case, for example, with HRiD_w3,4 (Figures 2D, 3C), where the power of the analysis was reduced, although we still detected positive selection.

In our analyses, eight closely related but distinct breeds were treated as a single unit (metapopulation), so possible genomic differences between breeds, or overrepresentation of some families could influence our results. For example, the high frequency of certain haplotypes in a few breeds could lead to misidentification of selection signals. Therefore, we performed an MJN analysis for each selection signal to determine whether all breeds were equally represented in favourable haplotypes. The results presented in Supplementary Figure S1 show a fairly even distribution of breeds in the selected haplotypes, which is consistent with our assumption that the identified selection signals are most likely the result of a long-term adaptive response to the local (Mediterranean) environment and the applied production system. In contrast, the presence of short-term selection, either natural or artificial, would result in “private” haplotypes occurring only in one or more breeds. Particular attention was also paid to the observed haplotype frequency distribution between “continental” and “island” breeds (see Supplementary Figure S1). While we were able to identify major haplotypes subject to selection in most signals, there were three signals (mapped from 32.20 to 32.80 Mb, from 42.50 to 43.00 Mb, and from 110.10 to 110.80 Mb) for which it was not clear which haplotype is selected, calling into question their identification as less reliable.

In summary, we identified 14 positive selection signals (12 regions) with a total of 34 annotated genes, with high repeatability (86%), as our 12 identified selection signals were also confirmed in other studies with sheep. Our novel approach HRiD identifies positive selection signals by searching for genomic regions that exhibit a sudden decrease in haplotype richness. Our results show that HRiD offers an interesting possibility to be used complementary to the eROHi, iHS and nSL approaches or when only males are genotyped, which is often the case in livestock where genomic breeding value estimates are routinely performed for males. Furthermore, we have shown that phylogenetic analyses, such as the Median-joining network, can provide useful additional information in the analysis of haplotypes identified as selection signals, either in terms of the ancestral or derived status of the advantageous selected haplotypes or by controlling for the potential confounding caused by population structure that may occur in the analysis of metapopulations. Overall, our results highlight the importance of the X-chromosome in the adaptive architecture of domestic ruminants, while our novel HRiD approach opens new avenues for research.

## Data Availability

The data presented in the study are deposited in the Dryad repository, accession number/link: https://doi.org/10.5061/dryad.hdr7sqvkk.
