# Supplementary material for: Identification of Selection Signals on the X-Chromosome in East Adriatic Sheep: A New Complementary Approach
Source: Front Genet. 2022 Apr 11;13:887582. doi: 10.3389/fgene.2022.887582 (PMC9126029; doi:10.3389/fgene.2022.887582)

**Supplementary Figure 1.** Median-joining network showing the phylogenetic relationship between haplotypes for all mapped selection signals with respect to their breed origin. The size of the haplotypes (number of SNPs) is indicated in parentheses.


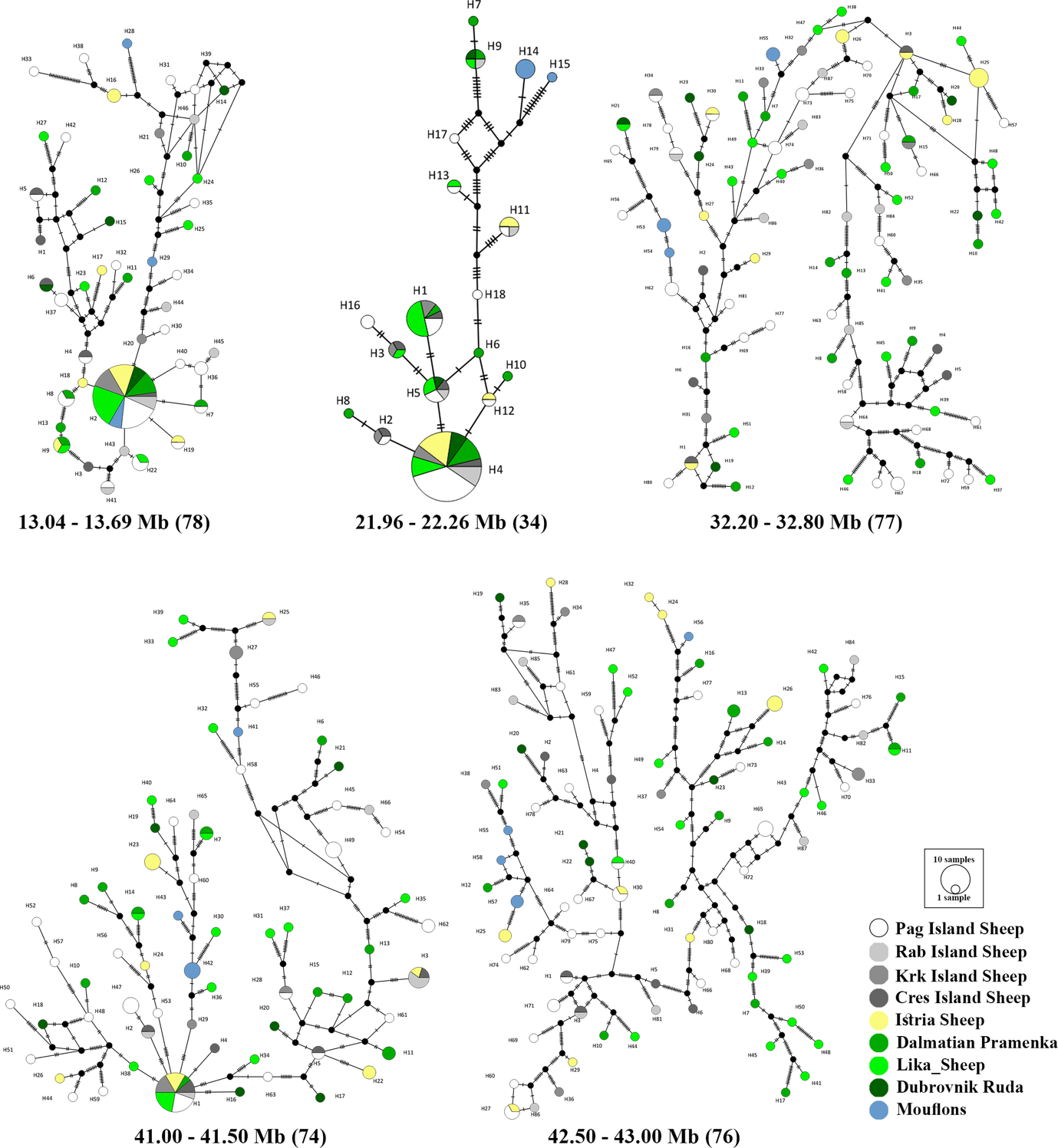


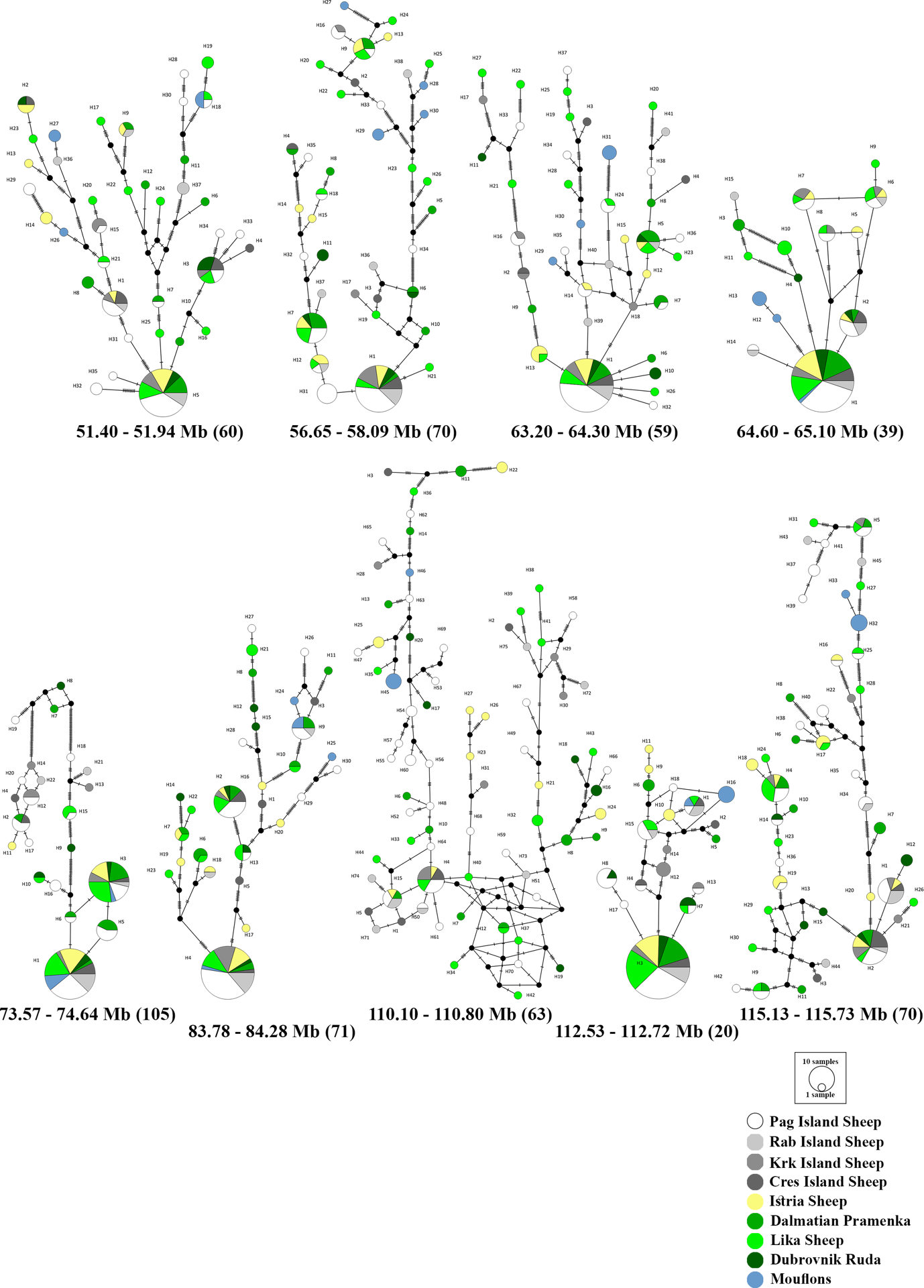

Supplement: Supplementary file 1 [file DataSheet1.zip › Supplementary_Material/Supplementary Figure S1.docx]
