# Supplementary material for: Identification of Selection Signals on the X-Chromosome in East Adriatic Sheep: A New Complementary Approach
Source: Front Genet. 2022 Apr 11;13:887582. doi: 10.3389/fgene.2022.887582 (PMC9126029; doi:10.3389/fgene.2022.887582)
Supplement: Supplementary file 1 [file DataSheet1.zip › Supplementary_Material/Supplementary File S1.docx]

**Supplementary File 1.** R script for recoding the VCF file so that the ancestral allele is the reference and the derived allele is the alternative (recodeVCF.R).

# Supplementary File 1. - R script to recode VCF file: ancestral allele - reference allele (REF column in VCF)

# derived allele - alternative allele (ALT column in VCF)

# Comments and information about the scripts (which are not executed) are prefixed with a hash sign (#)

# The information about the most frequent allele at each SNP in the mouflons was used to define the ancestral information

# The use of ancestral information (ancestral versus derived allele) is the preferred option in the iHS and nSL approach

#####################################

# #

# Recode VCF #

# #

#####################################

# Define the working directory where the input files are located (1. map file, 2. ancestral information file and 3. VCF file)

# Input of a map file (plink standard format)

mapa <- read.table(file = '220_chrX_ociscen.map',header = FALSE)

mapa <- mapa[,c(2,4)] # keeping only SNP name and bp position

colnames(mapa) <- c('SNPname','bp')

# Input of a previously determined ancestral allele for each SNP (for 17062 out of 18983 SNPs)

A_aleli <- read.csv('Ainf_mufloni.csv')

# Merging SNPs with their ancestral allele

A_aleli <- cbind(mapa,A_aleli$A_alel)

A_aleli <- subset(A_aleli,A_aleli$A_alel!=0) # If for a SNP mouflons where miss genotyped (1921 SNPs; no ancestral information provided), the initial information as ancestral were kept (the more frequent allele is the ancestral in that case)

# Subset of SNPs for which ancestral information was determined

snps <- as.data.frame(A_aleli$SNPname)

colnames(snps) <- 'ID'

# Loading the package 'dplyr' into R environment

library("dplyr")

# Input of a VCF file without description lines (skip = 5)

# IMPORTANT - first the character # must be removed manually before the beginning of the first column (in this case (skip = 5) remove # at the beginning of the 6th line) (#CHROM ---> CHROM)

vcf <- read.table('220_chrX_ociscen.vcf',skip = 5,header = TRUE)

vcf <- merge(vcf,snps,by = 'ID')

vcf <- arrange(vcf, POS) # this is were dplyr package is needed only (arrange function)

vcf <- vcf[,c(2,3,1,4:ncol(vcf))]

# Input of the ancestral information in INFO column of VCF file

vcf$INFO <- A_aleli$`A_aleli$A_alel`

# Recode VCF (REF column = ancestral allel, ALT column = derived allel)

refVCF <- NULL

n <- as.numeric(nrow(vcf)) # n = number of SNPs with ancestral information

c <- ncol(vcf) - 9 # c = number of individuals

for (i in 1:n) {

rr <- as.data.frame(vcf[i, ])

if(rr$ALT==rr$INFO){

rr$REF <- vcf[i,5] # 5 = ordinal number of referent column

rr$ALT <- vcf[i,4] # 4 = ordinal number of alternative column

for(j in 1:c) {

rr[1,9+j] <- ifelse(rr[1,9+j]=='0|1','1|0',

ifelse(rr[1,9+j]=='1|0','0|1',

ifelse(rr[1,9+j]=='1|1','0|0',

ifelse(rr[1,9+j]=='0|0','1|1'))))

}

}

refVCF <- rbind(refVCF,rr)

}

# refVCF = results

# Export VCF-a file with REF column = ancestral alel

write.table(refVCF,"recodedVCF.vcf",quote = FALSE,sep = ' ',row.names = FALSE)

# Finally, in this recoded VCF file, the next 5 lines must be added manually at the beginning (which were previously deleted) and the # sign at the beginning of the sixth line (CHROM ---> #CHROM)

# Below is an example of how the first 6th lines of the VCF file look like in our case:

##fileformat=VCFv4.1

##fileDate=05102021_09h42m21s

##source=SHAPEIT2.v904

##log_file=shapeit_05102021_09h42m21s_74e2d5ee-0c1f-4c37-8884-d907b32534b0.log

##FORMAT=<ID=GT,Number=1,Type=String,Description="Phased Genotype">

#CHROM POS ID REF ALT QUAL FILTER INFO FORMAT C156 C158 C159 C160... (title columns)
