# Supplementary material for: Identification of Selection Signals on the X-Chromosome in East Adriatic Sheep: A New Complementary Approach
Source: Front Genet. 2022 Apr 11;13:887582. doi: 10.3389/fgene.2022.887582 (PMC9126029; doi:10.3389/fgene.2022.887582)
Supplement: Supplementary file 1 [file DataSheet1.zip › Supplementary_Material/Supplementary File S2.docx]

**Supplementary File 2.** R script for calculating Haplotype Richness Drop values (HRiD.R).

# Supplementary File 2. - R script for HRiD estimation on chromosome X without PAR

# Comments and information about the scripts (which are not executed) are preceded by a hash sign (#)

# PAR has been previously excluded (from 0 to 7.04 Mb)

# A novel approach for identifying positive selection signals using male individuals (haplotypes) on chromosome X

# The sliding window approach is used to search for regions/windows where the decrease in haplotype richness is significant

# nh = Effective number of haplotypes

# nh = 1/sum(squared frequency of each haplotype)

# HRiD = Haplotype Richness Drop

# HRiD[wi+1] = (nh[wi] + nh[wi+2]) / 2*nh[wi+1]

#####################################

# #

# HRiD estimation #

# #

#####################################

# Define the working directory where the input file is located (VCF file)

# Input of a VCF file without description lines (skip = 5)

# IMPORTANT - first the character # must be removed manually before the beginning of the first column (in this case (skip = 5) remove # at the beginning of the 6th line) (#CHROM ---> CHROM)

vcf <- read.table('chrX_muske_onlyhapSNPs.vcf',skip = 5,header = TRUE)

# Loop for nh estimation of each window

# The size of the window is 70 SNPs, with a slide of 35 SNPs (both are editable)

geno <- vcf[,-1:-9] # First 9 columns were excluded in order to get only the genotypic information

geno <- as.data.frame(t(geno))

vcf$POS <- vcf$POS/1000000

geno <- rbind(geno,vcf$POS)

t<-1

i <- 70 # Here you can change the size of the window (70)

incidence <- NULL

start.Index <- NULL

end.Index <- NULL

start.position <- NULL

end.position <- NULL

o <- NULL

freq <- NULL

eff_N_alel <- NULL

while (i<=nrow(vcf)){

k <- geno[-nrow(geno),t:i]

w <- unique(k)

start.pos <- geno[nrow(geno),t]

start.position <- c(start.position,start.pos)

end.pos <- geno[nrow(geno),i]

end.position <- c(end.position,end.pos)

start.Index <- c(start.Index,t)

end.Index <- c(end.Index,i)

for (s in 1:nrow(w)) {

q <- w[s,]

for (y in 1:nrow(k)) {

a <- k[y,]

if(all(a==q)){

o <- rbind(o,a)

}

}

aa <- nrow(o)/nrow(k)

freq <- c(freq,aa)

o <- NULL

}

eff <- 1/sum(freq^2)

eff_N_alel <- c(eff_N_alel,eff)

incidence <- c(incidence,nrow(w))

HRiD <- as.data.frame(cbind(start.Index,start.position,end.Index,end.position,incidence,eff_N_alel))

t <- t+35 # Here you can change the size of the slide (35)

i <- i+35 # Here you can change the size of the slide (35)

freq <- NULL

}

# The last 23 SNPs were not estimated because there were not enough of them to construct a window (35 previous + 35 new)

HRiD <- as.data.frame(lapply(HRiD,as.numeric))

# Loop for HRiD estimation of each window (formula is modified for the first and the last window)

e <- 1

r <- 2

z <- 3

delta_Eff <- NULL

while (z <= nrow(HRiD)) {

w1 <- HRiD[e,6]

w2 <- HRiD[r,6]

w3 <- HRiD[z,6]

delta <- (w1 + w3)/(2 * w2)

if(e==1){

first <- w2/w1 # First window

delta_Eff <- c(delta_Eff,first)

}

delta_Eff <- c(delta_Eff,delta)

if(z==nrow(HRiD)){

last <- w2/w3 # Last window

delta_Eff <- c(delta_Eff,last)

}

e <- e + 1

r <- r + 1

z <- z + 1

}

HRiD$HRiD <- delta_Eff

# Normalisation of HRiD values

mean_HRiD <- mean(HRiD$HRiD)

sd_HRiD <- sd(HRiD$HRiD)

HRiD$Z_value <- (HRiD$HRiD - mean_HRiD)/sd_HRiD

# P value and -log(P) values

HRiD$P_value <- pnorm(-(HRiD$Z_value)) # One-sided test

HRiD$LogPValue <- -log10(HRiD$P_value)
