# Supplementary material for: Identification of Selection Signals on the X-Chromosome in East Adriatic Sheep: A New Complementary Approach
Source: Front Genet. 2022 Apr 11;13:887582. doi: 10.3389/fgene.2022.887582 (PMC9126029; doi:10.3389/fgene.2022.887582)
Supplement: Supplementary file 1 [file DataSheet1.zip › Supplementary_Material/Supplementary Table S1.docx]

**Supplementary Table 1**. Comparison of candidate regions mapped as selection signals on the X-chromosome in East Adriatic sheep with other sheep studies.

| Candidate region (Mb) | Overlapped signal (Mb) | Candidate genes | Reference |
| --- | --- | --- | --- |
| 13.04-13.69 | 13.20-13.60 | *CA5B*, *ZRSR2*, *AP1S2*, *GRPR* | Chen et al. (2018) |
| 21.96-22.26 | – |  |  |
| 32.20-32.80 | 31.98-32.38 | – | Zhu et al. (2015) |
|  | 32.40-32.60 | – | Chen et al. (2018) |
|  | 32.10-32.40 | – | Liu et al. (2016) |
| 41.00-43.00 | 41.36-41.76 | *EFHC2* | Zhu et al. (2015) |
|  | 41.20-41.40 | *EFHC2* | Chen et al. (2018) |
|  | 38.94-44.20 | *NDP, EFHC2* | Cezarini et al. (2022) |
| 51.40-51.94 | 51.17-51.91 | *SHROOM4*, *DGKK*, *CCNB3* | Zhu et al. (2015) |
|  | 51.18-51.56 | *SHROOM4* | Zhu et al. (2019) |
| 56.64-58.09 | 57.4-58.0 | *OPHN1*, *YIPF6* | Chen et al. (2018) |
|  | 56.50-58.00 | *AR*, *OPHN1*, *YIPF6* | Liu et al. (2016) |
|  | 55.78-58.33 | *AR*, *OPHN1*, *YIPF6* | Cezarini et al. (2022) |
|  | 55.79-58.35 | *AR*, *OPHN1*, *YIPF6* | Manzari et al. (2019) |
| 63.20-65.10 | 63.09-63.46 | *RLIM*, *KIAA2022*, *ABCB7*, MAGT1, ATRX, FGF16 | Zhu et al. (2019) |
|  | 64.80-65.00 | *ATRX, FGF16* | Chen et al. (2018) |
|  | 64.95-65.05 | *ATRX, FGF16* | Kardos et al. (2015) |
| 73.57-74.54 | 73.69-74.09 | *CHM*, *DACH2* | Zhu et al. (2015) |
|  | 73.44-73.94 | *CHM*, *DACH2* | Zhu et al. (2019) |
| 83.78-84.28 | – |  |  |
| 110.10-110.80 | 110.20-110.40 | *DOCK11* | Chen et al. (2018) |
| 112.53-112.72 | 112.66-113.06 | *PLS3* | Zhu et al. (2015) |
| 115.30-115.73 | – |  |  |
